# Metabolic reprogram and T cell differentiation in inflammation: current evidence and future perspectives

**DOI:** 10.1038/s41420-025-02403-1

**Published:** 2025-03-28

**Authors:** Yuxin Shi, Hao Zhang, Changhong Miao

**Affiliations:** 1 Department of Anesthesiology, Zhongshan Hospital, Fudan University, Shanghai, China; 2 Shanghai Key Laboratory of Perioperative Stress and Protection, Shanghai, China; 3 Department of Anesthesiology, Shanghai Medical College, Fudan University, Shanghai, China

**Keywords:** Inflammation, Lymphocytes

## Abstract

T cell metabolism and differentiation significantly shape the initiation, progression, and resolution of inflammatory responses. Upon activation, T cells undergo extensive metabolic shifts to meet distinct functional demands across various inflammatory stages. These metabolic alterations are not only critical for defining different T cell subsets, but also for sustaining their activity in inflammatory environments. Key signaling pathways—including mTOR, HIF-1α, and AMPK regulate these metabolic adaptions, linking cellular energy states with T cell fate decisions. Insights into the metabolic regulation of T cells offer potential therapeutic strategies to manipulate T cell function, with implications for treating autoimmune diseases, chronic inflammation, and cancer by targeting specific metabolic pathways.

## Facts

1. Different metabolic pathways are crucial for the differentiation and function of T cell subsets during inflammatory responses.
2. Glycolysis promotes effector T cell function, while regulatory T cells prefer oxidation metabolism and fatty acid oxidation.
3. Metabolic reprogramming-targeted therapies have shown effectiveness in treating inflammatory diseases.

## Open questions

1. How does the metabolic reprogramming influence memory T cell formation and maintenance during and after inflammation?
2. Is it possible that metabolic modulation selectively target pathogenic T cell subsets in autoimmune diseases?
3. Emerging evidences shed light on how metabolic changes affect memory T cell subsets in tumor microenvironments. How do metabolic changes during the differentiation and activation of CD4^+^ T cells affect their long-term function, including susceptibility to exhaustion and their ability to maintain immune surveillance?

## Introduction

The inflammatory response serves as a core defense mechanism against infection and tissue damage, driven by a multifaceted network of immune cell activation. T cells integral to modulating inflammation are crucial in regulating immune responses, as part of the adaptive immune system. These cells exhibit notable plasticity, undergoing swift activation, proliferation, and differentiation upon exposure to inflammatory stimuli [1]. This dynamic behavior is tightly regulated through complex signaling pathways, transcription factors, and substantial metabolic reprogramming, which adjusts to meet the varying demands of distinct T cell subsets.

Metabolic reprogramming in T cells is fundamental to their function and differentiation [2]. Upon activation, naïve T cells transition from a quiescent state, driven by oxidative phosphorylation (OXPHOS), to a glycolytic phenotype, which sustains the heightened energy and biosynthetic demands required for rapid proliferation and effector activity. Distinct T cell subsets, including Th1, Th2, Th17 effector T cells (Teffs), and regulatory T cells (Tregs), display unique metabolic signatures [3–6]. Emerging evidence highlights that metabolic shifts in T cells influence not only their physiological roles but also contribute to the pathogenesis and progression of various inflammatory diseases. Throughout inflammation, T cells dynamically modulate their metabolism to align with varying immune demands [7].

Inflammatory conditions actively shape metabolic pathways, thereby directing T cell differentiation and function [8, 9]. Pro-inflammatory cytokines, including IL-6, IL-12, and TNF-α drive glycolysis and anabolic processes, promoting Teff differentiation [6]. In contrast, anti-inflammatory cytokines such as TGF-β and IL-10 promote oxidative metabolism and mitochondrial respiration, supporting Treg development and function [10]. These metabolic adaptions are intricately connected to critical signaling pathways like mTOR, AMPK, and HIF-1α, which integrate nutrient status and inflammatory signals [11, 12].

Understanding the interaction between inflammation and T cell metabolism is vital for developing therapeutic interventions for various diseases. Dysregulations in T cell metabolic processes are associated with the development of autoimmune disorders, persistent inflammatory conditions, and cancer, where imbalances in T cell activation or regulatory dysfunctions of T cells contribute to disease progression [13]. Modulating these metabolic pathways to influence T cell behavior offers a promising approach for advancing immunotherapeutic treatments.

## Metabolic programming and regulation of T cells

### The basics of T cell metabolism

T cells play an essential role in the adaptive immune response, with their functions dependent on highly regulated metabolic pathways. T cell metabolism encompasses the processes through which these cells generate and manage energy to sustain their survival, expansion, differentiation, and functional activities [14]. Throughout the transitions between resting, activation, proliferation, and effector phases, T cells undergo profound metabolic shifts, allowing them to meet the changing bioenergetic and biosynthetic needs correlated with each stage.

Resting T cells maintain a low metabolic profile, relying predominantly on OXPHOS for energy. Simultaneously, fatty acid oxidation (FAO) provides acetyl-CoA, which fuels the tricarboxylic acid (TCA) cycle to supply substrates for OXPHOS. Upon activation by antigen-presenting cells (APCs) through T cell receptor (TCR) engagement, T cell metabolic requirements surge dramatically [15, 16]. Activated T cells, similar to cancer cells, shift towards elevated aerobic glycolysis, resulting in increased lactate production. Additionally, the glutamine and serine metabolic pathways play considerable role in activated T cells; glutamine contributes intermediates for the TCA cycle, while the serine pathway supports one-carbon metabolism, promoting nucleotide synthesis and cell proliferation.

**Fig. 1:**
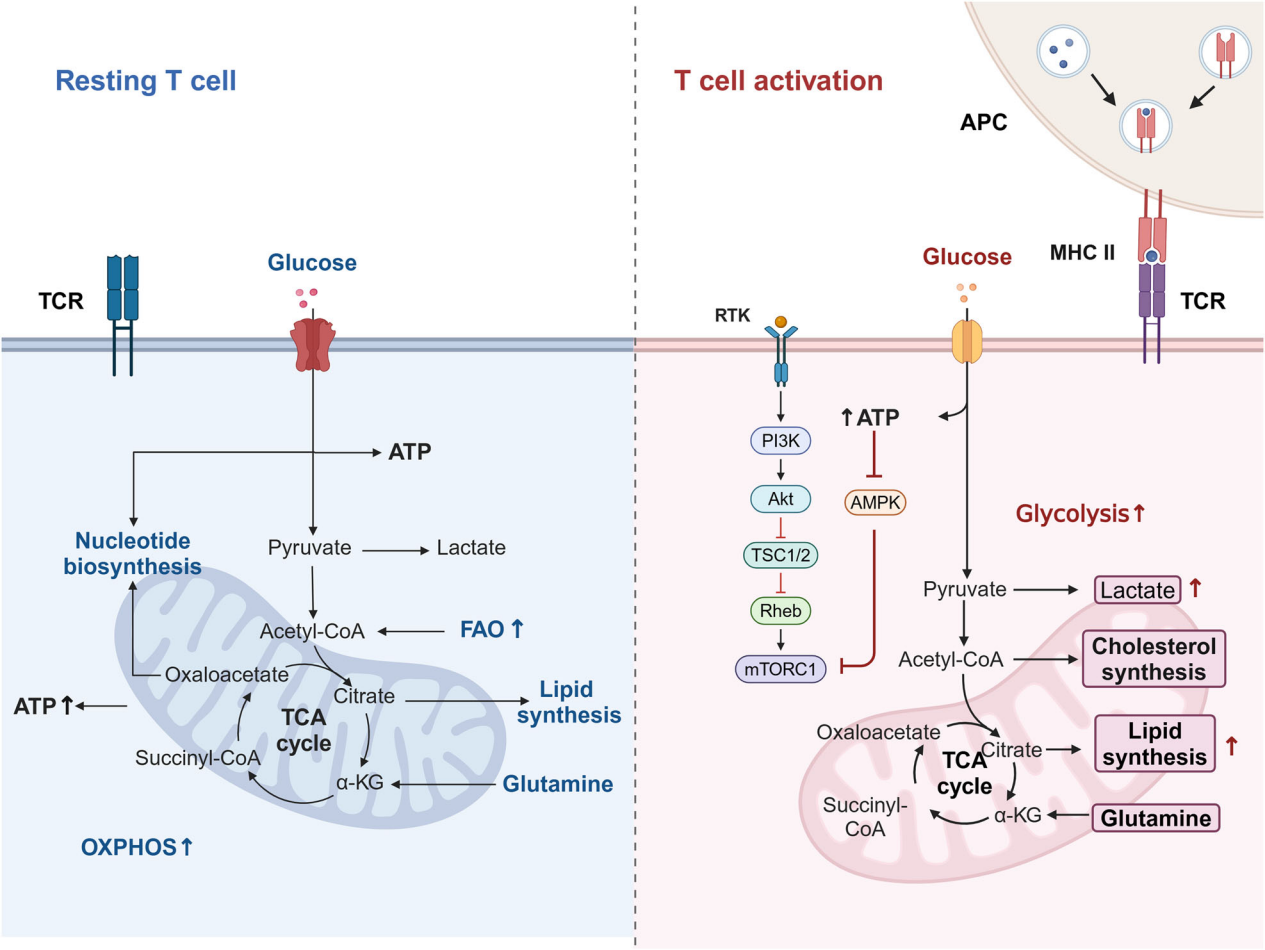
Metabolic pathway shifts in T cells. Dynamic changes occur in T cell metabolism upon T cell activation, highlighting the shift from OXPHOS to glycolysis during activation. This metabolic reprogramming supports the rapid proliferation and increased functionality of effector T cells in response to inflammatory signals. Resting T cells, shown on the left side, predominantly utilize OXPHOS as their main energy source, relying on the mitochondria for efficient ATP production in a low-energy demand state. On the right side, activated T cells exhibit a metabolic shift towards glycolysis, which enables T cells to generate ATP more rapidly to meet the increased energy demands of proliferation and effector functions.

Teffs, as the fully differentiated stage of activated T cells, are responsible for mediating immune responses. Their rapid response to pathogens is sustained through an increased reliance on glycolysis [17]. Concurrently, lipid biosynthesis is enhanced, while FAO is suppressed, supporting cell membrane expansion and promoting signal transduction. Unlike Teffs, memory T cells (Tmem) and Tregs, which are essential for preserving immune homeostasis and modulating inflammatory responses, primarily depend on OXPHOS and FAO to maintain their long-term functions [18, 19] (Fig. 1).

### The relationship between metabolic pathway regulation and T cell function

T cell functionality is intricately regulated through complex metabolic networks that integrate environment cues with cellular demands. These metabolic pathways precisely orchestrate T cell activation, proliferation, differentiation, and effector functions through complex signaling networks. The dynamic interplay between metabolic programming and cellular function ensures appropriate immune responses while maintaining homeostasis.

During T cell activation, enhanced glycolysis supports Teff functions by providing rapid ATP generation and biosynthetic intermediates [17]. This metabolic shift happens through PI3K-Akt-mTOR pathway activation, leading to increased glucose transporter (GLUT1) expression and glucose uptake [20]. TCR signaling, initiates this metabolic reprogramming through downstream effectors including Lck, ZAP-70, and LAT [21]. The resultant enhanced glycolytic flux directly supports rapid proliferation. Consequently, this metabolic programming enables sustained IL-2 and other effector cytokine production through NFAT and NF-κB activation [22].

Tmem formation and maintenance critically depend on OXPHOS. This metabolic program provides stable ATP production through efficient substrate utilization, while FAO generates acetyl-CoA to maintain metabolic homeostasis [23]. AMPK activation triggers PGC-1α expression, enhancing mitochondrial biogenesis and OXPHOS capacity [24]. The calcium-dependent signaling through NFAT coordinates with AMPK to establish metabolic memory programming [25]. This metabolic state promotes long-term survival and functional persistence of memory T cells through optimized energy utilization.

Lipid metabolic pathways direct T cell lineage commitment through multiple mechanisms. FAO supports Treg development and function, while de novo fatty acid synthesis promotes Th17 differentiation [26]. Costimulatory signals through CD28 amplify these metabolic programs via PI3K-Akt signaling, which suppresses FOXO transcription factors and enhances mTOR activation [27]. The peroxisome proliferator-activated receptor (PPAR)γ-mediated transcriptional network integrates with mTOR/AMPK signaling to establish subset-specific lipid utilization patterns, ultimately influencing T cell fate decisions through the regulation of key transcription factors like T-bet, GATA3, RORγt, and Foxp3 [28].

Glutamine metabolism supports proliferation through anaplerotic reactions, while tryptophan metabolism influences the Treg/Th17 balance [29]. The LAT1 amino acid transporter system coordinates with mTOR activation to drive metabolic reprogramming. This process is further regulated by NF-κB signaling, which is essential for the differentiation of Th1 and Th17 cells through the upregulation of inflammatory genes [30]. The integrated network of metabolic programming and signaling pathways enables precise control of T cell responses in various immunological contexts.

## The impact of the inflammatory environment on T cell differentiation

**Fig. 2:**
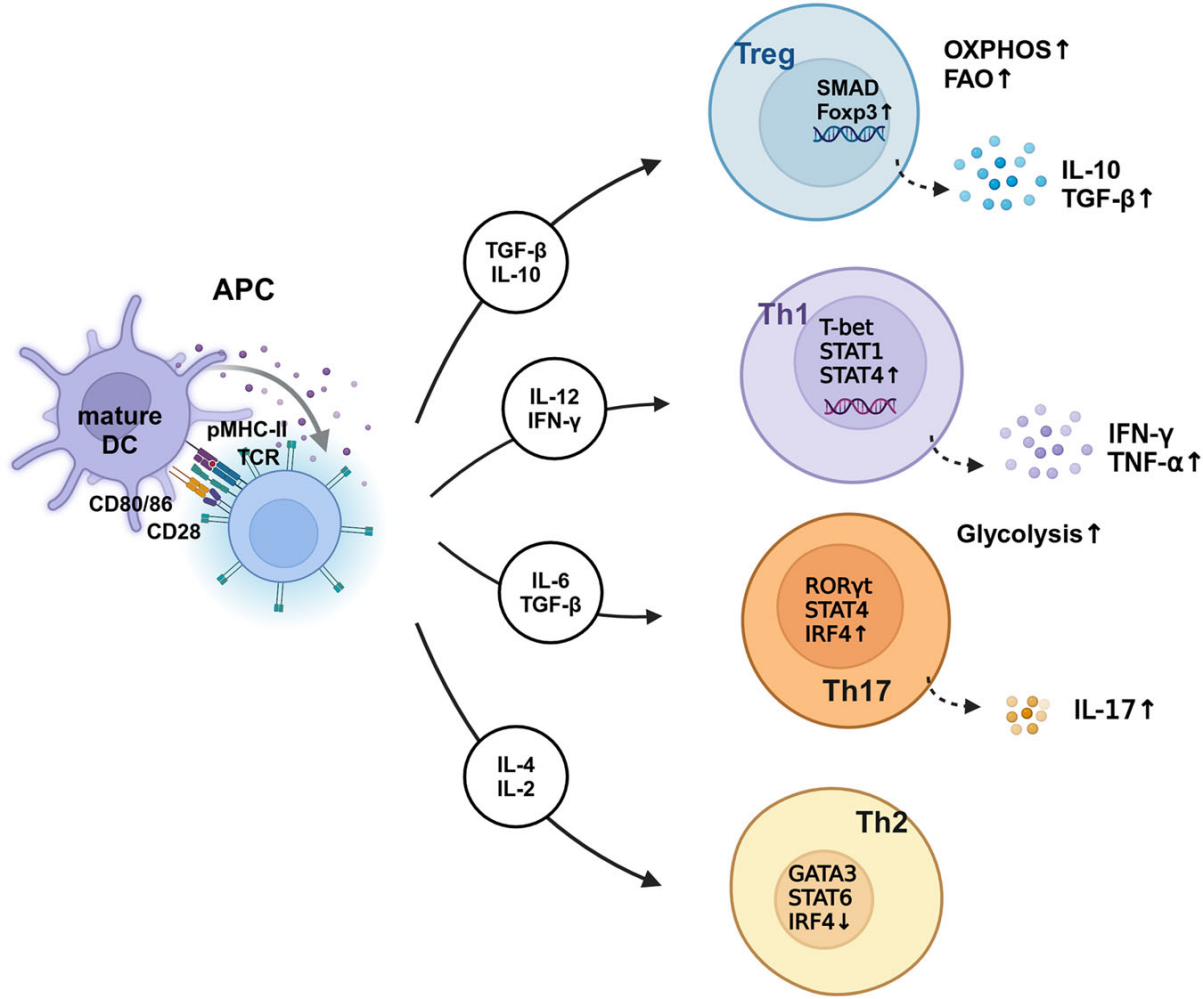
Differentiation and metabolic profiles of T cell subsets under inflammatory conditions. This figure outlines the differentiation of naive T cells into distinct subsets, such as Th1, Th2, Th17, and Tregs, under the influence of inflammatory and metabolic signals. Upon activation, the naive T cell can differentiate into various effector or regulatory subsets based on the surrounding cytokine milieu and the metabolic environment. Each subset of T cells exhibits a unique metabolic profile that supports its specific functional demands. Th1, Th17, and Th2 cells predominantly rely on aerobic glycolysis, while Tregs prefer OXPHOS and FAO. Metabolic programs are differently linked to T cell lineage commitment and their distinct immune functions.

T cells development originates in the thymus, where distinct cytokine environments drive differentiation into various functional subtypes, each integral to specific immune responses [31]. This developmental process progresses through three stages: naïve (or resting), effector (or activated), and memory T cells. Naïve T cells become activated upon antigen recognition through TCR engagement with major histocompatibility complex (MHC) molecules [32]. Helper T cells (Th cells) initially exist as Th0 precursors, differentiating into specialized subtypes in response to distinct cytokine cues [33] (Fig. 2). CD8^+^ T cells, activated by APCs via MHC class I molecules, differentiate into cytotoxic T lymphocytes (CTLs), which eliminate pathogen-infected or tumor cells through the release of perforin and granzymes [34, 35]. Tmems, derived from either Th cells or cytotoxic T cells (Tc cells), rapidly mount a secondary immune response upon subsequent antigen exposure.

### Cytokine microenvironment

Cytokine fluctuations considerably influence T cell differentiation during inflammation. Inflammatory environment stimulates innate immune cells, including dendritic cells (DCs), macrophages, and monocytes to secrete cytokines, such as IL-1, IL-6, IL-12, TNF-α, and IFN-γ, which in turn activate and modulate adaptive immune responses [36]. These cytokines engage specific receptors on T cells, triggering signaling cascades that guide differentiation into various T cell subsets. Additionally, anti-inflammatory cytokines like IL-10 and TGF-β are produced to mitigate excessive immune activity, facilitate tissue repair, and maintain immune homeostasis.

In acute inflammation, elevated IL-12 and IFN-γ levels induce Th1 differentiation, amplifying cell-mediated immune responses to efficiently eliminate viral infections and intracellular pathogens [25]. In contrast, Th17 cells dominate in chronic inflammation and autoimmune conditions [37, 38]. Persistent IL-6 and TGF-β signaling drive Th17 differentiation, perpetuating chronic inflammatory responses [39]. During microbiota-driven differentiation, Th17 cells arise from naïve CD4^+^ T cells via RORγt, producing IL-17A and IL-17F, which maintain gut homeostasis [40]. In murine models, intrinsic IRF5 in T cells boosts Th1 and Th17 cytokine production, suppresses Th2 cytokines, and promotes T follicular helper (Tfh) cell differentiation in vivo [5, 25, 41, 42]. Within the tumor microenvironment (TME) and in chronic inflammatory settings, elevated TGF-β and IL-10 suppress effector T cell activation while expanding Tregs, fostering an immunosuppressive milieu [43].

### Costimulatory signals

Costimulatory signals, originating predominantly from APCs, play a fundamental role in driving T cell differentiation amid inflammation conditions [44]. Within this context, APCs become activated through exposure to pathogen-associated molecular patterns (PAMPs) or damage-associated molecular patterns, triggering the upregulation of costimulatory molecules [45]. The heightened presence of these signals intensifies T cell activation and differentiation, promoting a more efficient immune response against pathogens [46]. In atopic dermatitis, keratinocytes from the compromised skin barrier release cytokines such as thymic stromal lymphopoietin (TSLP), IL-25, and IL-33, which activate APCs to express OX40L [47]. This activation subsequently drives the expansion of specific T cell subsets, increasing the production of IFN-γ, IL-17, or IL-22, thereby intensifying the Th1, Th2, and Th17/22 pathways [47]. This cascade results in the persistent accumulation and heightened activity of effector and memory Th1, Th2, Th17, and Th22 cells [48]. In autoimmune conditions like rheumatoid arthritis and type 1 diabetes, the single nucleotide polymorphism (SNP) rs117701653 has been implicated in regulating ICOS expression by altering allelic affinity for the inhibitory chromatin regulator SMCHD1 [49]. This modulation accelerates the generation of peripheral helper T (Tph) cells, exacerbating disease progression.

### Microbiota and inflammation

The gut microbiota is integral to sustaining intestinal immune balance and modulating T cell differentiation [50]. Metabolites such as butyrate, propionate, and acetate are generated by commensal bacteria through the fermentation of dietary fiber, influencing Treg differentiation via epigenetic mechanisms, notably the inhibition of histone deacetylases (HDACs) [51]. Studies have demonstrated that SCFAs enhance the formation and stability of Foxp3^+^ Tregs within inflammatory contexts by engaging G-protein-coupled receptors (GPCRs) including GPR43 and GPR109A [52]. This engagement triggers distinct signaling cascades that preserve immune tolerance and mitigate intestinal inflammation.

Microbiota dysbiosis, on the other hand, is closely associated with Th17 cell expansion and an amplification of Th1/Th17-mediated inflammatory responses [53, 54]. Certain gut commensals, such as *Bacteroides fragilis* and *Clostridia*, remarkably contribute to the induction of Tregs [55]. Conversely, other commensals like segmented filamentous bacteria (SFB) engage with intestinal epithelial cells, promoting Th17 cell differentiation and accumulation in the lamina propria of small intestine [56]. Furthermore, under inflammatory conditions, the microbiota’s influence on T cell differentiation is intricately intertwined with the host’s immune profile and disease progression [57, 58]. In patients with inflammatory bowel disease (IBD), substantial alterations in the gut microbiota composition and function promote the expansion of pro-inflammatory Th1 and Th17 cells, intensifying intestinal inflammation [55, 59].

## Metabolic dependency of T cell differentiation in inflammatory conditions

**Fig. 3:**
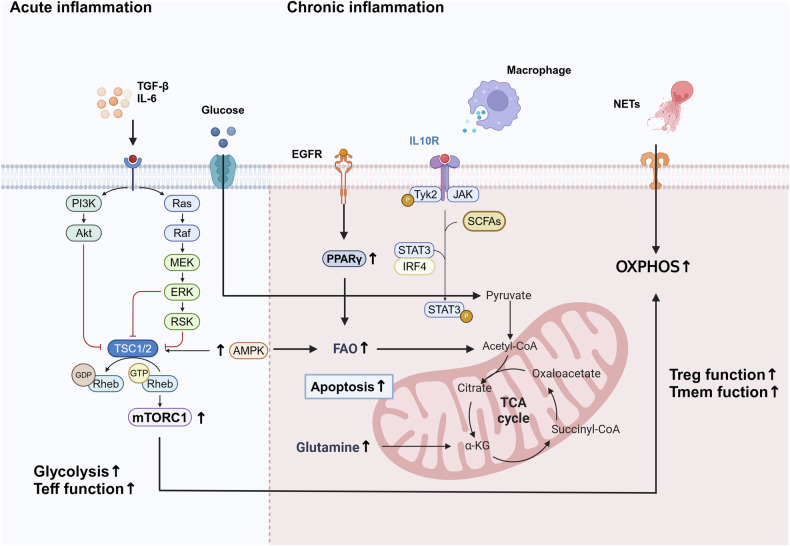
Impact of the inflammatory microenvironment on T cell metabolism and function. During the progression of inflammation, T cell metabolism undergoes reprogramming in response to alterations in cytokines and the microenvironment. In acute conditions, pro-inflammatory cytokines drive T cell activation and promote glycolytic metabolism, while promoting OXPHOS and FAO in chronic conditions. These metabolic adaptations support T cell effector functions or immune regulation. Additionally, the interplay between T cells and other immune cells, such as macrophages and DCs, is depicted, emphasizing the metabolic crosstalk that shapes immune responses.

The regulation of metabolic pathways plays a decisive role in shaping T cell functionality during differentiation[60]. Distinct metabolic states are tightly linked to the specialization of T cell subsets [61, 62]. Inflammatory conditions, trigger differentiation into various T cell subsets, accompanied by profound metabolic shifts [63, 64]. Cytokines and metabolites present in the inflammatory environment further modulate these differentiation processes [25, 65–67]. Such metabolic reprogramming enables T cells to swiftly adapt to diverse immune microenvironments, ensuring rapid and effective responses during inflammation (Fig. 3).

## Glycolysis and T cell differentiation in inflammatory status

Glycolysis serves as a key metabolic pathway for T cell activation, proliferation, and differentiation in inflammatory conditions [68]. During the differentiation of naïve CD4^+^ T cells into inflammatory Teffs, a metabolic shift from OXPHOS to predominantly glycolysis and glutamine metabolism occurs [69]. Metabolic assessments reveal that pyruvate dehydrogenase (PDH) acts as a central node linking glycolysis and oxidative metabolism in T cells. Inhibiting or knocking down PDHK1 selectively impairs Th17 cells while augmenting Treg function [70]. In tumor-infiltrating Tregs, heightened glycolytic activity correlates with CCR6-CCL20 signaling [71]. Pro-inflammatory cytokines such as IL-6 and TGF-β enhance glycolysis in T cells via the phosphoinositide 3-kinase (PI3K)-Akt-mTOR signaling axis [20, 72]. mTOR, a primary regulator of glycolysis, not only facilitates glucose uptake and upregulates key glycolytic enzymes but also promotes Th17 differentiation and pathogenicity through downstream activation of HIF-1α [11, 73]. HIF-1α directly binds to RORγt, the transcription regulator of Th17 cells, enhancing its activity and promoting further Th17 differentiation and pro-inflammatory responses [11]. Inflammatory conditions are frequently accompanied by tissue hypoxia, which activates HIF-1α, promoting Teff differentiation. At the same time, HIF-1α can suppress Treg differentiation, contributing to the regulation of the Th17/Treg balance in inflammation [74, 75].

Regulation of glycolysis plays a central role in the pathogenesis of inflammatory diseases. In autoimmune disorders such as rheumatoid arthritis and IBD, T cells within affected tissues display heightened glycolytic activity, leading to elevated production of pro-inflammatory cytokines [26]. Increased lactate concentrations in the microenvironment suppress OXPHOS while enhancing glycolysis, intensifying Teff activation [76]. RhoA activation in T cells accelerates metabolic pathways, including both glycolysis and OXPHOS, promoting Th2 differentiation and contributing to allergic airway inflammation [77].

### OXPHOS and T cell differentiation in inflammatory status

In contrast to Teffs, which primarily utilize glycolysis, Tregs depend on mitochondrial OXPHOS for energy production and the preservation of cellular homeostasis. During chronic inflammation or extended antigen stimulation, OXPHOS is essential for both the development and maintenance of Tregs and Tmem cells. In the immunosuppressive phase of sepsis and in hepatocellular carcinoma, neutrophil extracellular traps (NETs) drive CD4^+^ T cell differentiation into Tregs by upregulating OXPHOS, contributing to disease progression and tumor metastasis [78–80]. Moreover, mitochondrial dynamics, such as fusion and fission, notably affect OXPHOS efficiency and modulate T cell functions [81]. Mitochondrial fusion enhances OXPHOS activity, supporting Treg differentiation and function, whereas mitochondrial fission is linked to Teff differentiation [82]. Additionally, OXPHOS augments the anti-apoptotic properties and persistence of Th17 cells by inhibiting mitophagy, preventing the degradation of apoptosis-regulating factors [83].

During the differentiation of Th17 and Tregs, OXPHOS integrates with the mTOR, AMPK, and HIF-1α signaling pathways, establishing a complex metabolic regulatory network [84]. Inflammatory conditions frequently induce dysregulated energy metabolism, leading to AMPK activation [12]. mTORC1 signaling augments glycolysis in Teffs by inhibiting mitochondrial autophagy and promoting mitochondrial biogenesis, while AMPK activation enhances OXPHOS in Tregs through the stimulation of mitochondrial autophagy and FAO [28]. In the context of tumor progression and viral infection, Sirt3 modulates NAD^+^-dependent glycolysis caused by mitochondrial OXPHOS impairment, reprogramming Tfh differentiation via the mTOR-HIF1α-Bcl6 axis [85].

### FAO and T cell differentiation in inflammatory status

FAO plays a key role in regulating the balance between Tregs and Teffs by oxidizing long-chain fatty acids and modulating the production of metabolic intermediates [26, 79, 86]. Through enhanced mitochondrial FAO pathways, Tregs effectively suppress inflammation and maintain immune tolerance. Oxidized phospholipids can inhibit the upregulation of CD40 and the production of IL-1β, along with other pro-inflammatory cytokines, while simultaneously promoting IL-10 secretion by DCs [87]. Importantly, Th17 cells are closely associated with FAO [26]. Studies indicate that Th17 cells can preserve their pathogenicity and survival in inflammatory environments by adjusting lipid metabolic pathways, including fatty acid synthesis and oxidation. In chronic inflammation, alterations in SCFA metabolism drive the formation of the IRF4-STAT3 complex and increase STAT3 phosphorylation, promoting the differentiation of pathogenic Th17 cells [88].

In addition, FAO is essential for the differentiation and survival of CD8^+^ memory T (Tm) cells. Studies demonstrate that the metabolic profile of CD8^+^ Tm cells shifts from glycolysis to FAO, a transition that supports their longevity and sustained viability [81]. PPAR activation in CD8^+^ T cells promotes this metabolic transition, enhancing both their energy metabolism and effector capabilities [89]. This metabolic adaption becomes particularly meaningful in inflammatory environments, where reprogramming provides the necessary energy and substrates required for the differentiation into effector or memory cells [90].

### Amino acid metabolism and T cell differentiation in inflammatory status

In inflammatory conditions, immune cells like DCs and macrophages actively regulate amino acid metabolism within the microenvironment, exerting a direct influence on T cell fate [30, 91]. Glutamine metabolism, during the inflammatory response, supplies carbon skeletons for the TCA cycle, sustaining T cell proliferation and differentiation [92]. Evidence suggests that glutamine metabolism is critical for Teff function, while glutamine depletion shifts T cell differentiation towards Tregs [92]. In clear-cell renal cell carcinoma, macrophage-derived IL-23, promotes Treg expansion and elevates IL-10 and TGF-β expression, suppressing CTL-mediated tumor cell destruction [93]. In autoimmune diseases, dysregulated expression of the glutaminase enzyme GLS1 drives increased acetyl-CoA production, leading to enhanced histone acetylation at the Il17a promoter, thereby promoting Th17 and γδT17 cell differentiation [94]. In autoimmune hepatitis (AIH), GLS antagonists like JHU083 and DON not only attenuate T cell activation and reduce the Th1/Th17 ratio but also downregulate SLC7A5 mRNA expression, further dampening mTOR pathway activation [95, 96]. Leucine metabolism plays a dual regulatory role in mTOR signaling during T cell immune responses, where leucine deficiency reduces energy availability and promotes Tmem differentiation [97, 98].

IDO is abundantly expressed in specific immune cells, particularly within inflammatory environments, where it regulates tryptophan metabolism. Its metabolite, kynurenine, suppresses Teff function while promoting Treg differentiation through aryl hydrocarbon receptor (AhR) activation. Tryptophan depletion triggers general control nonderepressible 2 (GCN2)-mediated upregulation of SLC7A5 (LAT1), facilitating increased kynurenine uptake [30]. LAT1 is essential for the activation of pathogenic T cell subsets in inflammation. LAT1 depletion in mouse CD4^+^ T cells attenuates experimental arthritis and reduces IFN-γ and TNF-α-producing T cell differentiation [99, 100]. The interaction between IDO and AhR pathways is crucial for immunosuppression across tumor settings and chronic inflammatory diseases. Arginine, a key modulator of T cell proliferation, generates nitric oxide (NO), which influences the Th1/Th2 balance during inflammation. Decreased arginine levels or excessive NO production suppress T cell proliferation and promote Treg differentiation. In CLP mice, citrulline supplementation elevated plasma arginine and citrulline levels, leading to a decreased Treg proportion [101]. Under conditions of infection or acute inflammation, arginine depletion enhances Th1 differentiation, supporting antigen-specific immune responses [102].

## Targeting metabolic pathways in treating inflammatory diseases

T cell metabolism acts importantly in modulating inflammatory immune responses [103]. Advances in the understanding of the regulatory mechanisms of T cell metabolism have established metabolism as a central factor influencing T cell function [104, 105]. A table of clinically available targeted therapies has been compiled illustrating the role of metabolic modulation in inflammatory immunotherapy (Table 1). By either augmenting or suppressing T cell activity and differentiation, immune responses can be finely controlled. Emerging research has identified novel metabolic checkpoints, offering promising targets for immunotherapeutic interventions. Regulation of these metabolic checkpoints is crucial for controlling immune responses in inflammatory diseases.

**Table 1:** Specific agents targeting different metabolic modes that targeting inflammatory diseases.

Therapy	Mechanism of treatment	Effects on T cells	Target disease	References
Fasudil	Inhibits OXPHOS and glycolysis	Reduces Th2 cell differentiation	Allergic airway inflammation	[77]
Troglitazone	Enhances basal respiratory capacity and ATP production	Increases IL-5 and IL-13 in Th2 cells	Allergic asthma	[147]
NG52	Reduces glycolysis; promotes ROS accumulation	Inhibits Th17 cells development	Myocarditis	[106]
Itaconic acid salt (ITA)	Inhibits glycolysis and OXPHOS	Inhibits Th17 cells； promotes Tregs differentiation	EAE	[110]
Somatostatin (SST)	Reduces mitochondrial respiration	Inhibits Th1 and Th17 cells	EAE	[116]
MTHFD2i; LY345899	Inhibits glycolysis; promotes OXPHOS	Inhibits Th17 cells; promotes Tregs differentiation	EAE and IBD	[148]
Antimycin A	Inhibits glycolysis	Inhibits Th17 cells proliferation	EAE and IBD	[149]
NCA029	Promotes OXPHOS	Adjust the Th17/Treg imbalance	IBD	[115]
CpdA	Enhances the expression of HIF-1α in glycolysis	Promotes IL-10 in CD4^+^ T cells	IBD	[52]
UNC0642	Promotes OXPHOS and lipid membrane composition	Promotes Tregs expansion	IBD	[119]
DMαKG	Reduces glucose carbon flow in the TCA cycle; enhances OXPHOS	Restores Tregs function	IBD	[109]
AGK2, TM	Inhibits glycolysis and OXPHOS	Enhances T cell proliferation and effector function	IBD	[150]
Etoxomir, Ranolazine	Inhibits FAO	Reverses T cell anti-apoptotic and pro-inflammatory phenotype	IBD	[121]
10058-F4	Inhibits OXPHOS; promotes glycolysis	Diminishs the suppressive function of Tregs on Teffs	IBD and GvHD	[114]
TEPP-46	Inhibits glycolysis	Inhibits Tfh cell differentiation	SLE	[151]
Rapamycin, 2-DG	Inhibits fatty acid synthesis	Increases Th1	SLE	[152]
ABT-263	Upregulates OXPHOS	Protects Th17 cells from apoptosis	Melanoma	[83]
BCAA supplement	Promotes glucose uptake, glycolysis, and OXPHOS	Promotes effector function of CD8^+^ T cell	NSCLC	[134]
IACS-010759	Inhibits OXPHOS	Inhibits T cell growth	T-ALL	[111]
AS3385282、ASP7657	Upregulates glycolysis and OXPHOS	Restores CD8^+^ T cell function	Ovarian cancer	[24]
GW0742	Increases mitochondrial respiration	Restores CD8^+^ T cell mitochondrial function	Colorectal cancer and in situ glioma	[130]

### Strategic approaches in autoimmune disease

#### Targeting the glycolysis-PPP axis

Glycolysis functions as a key metabolic regulator that determines the balance between Teff and Treg differentiation. Treatment with NG52, a PDHK1 inhibitor, blocked glycolysis and led to ROS accumulation, thereby suppressing the CD4^+^ T cell and Th17 cell responses in patients with myocarditis [106]. In experimental autoimmune uveitis (EAU) mice, the lactylation of Ikzf1 at the lys164 site in CD4^+^ T cells is elevated and modulates Th17 differentiation. Decreased lactylation of CD4^+^ T cells inhibits Th17 differentiation and impedes the progression of EAU [107].

Transcriptomic and carbon tracing analyses reveal that activation of CD4^+^ T cells drives glycolysis, resulting in the production of extramitochondrial pyruvate, which is subsequently converted to acetyl-CoA via PDH [70]. Inhibitor of glycolysis with 2-DG, a classical glycolysis inhibitor, has been demonstrated to suppress Th17 development while promoting Treg induction [108]. Deficiency in transketolase (TKT), a pivotal enzyme in the non-oxidative pentose phosphate pathway (PPP), reduces glycolysis and increases oxidative stress, leading to enhanced fatty acid and amino acid catabolism, which subsequently boosts OXPHOS. Supplementation with α-KG has been shown to restore Treg function in TKT-deficient mice and alleviate autoimmune conditions [109]. In the murine experimental autoimmune encephalomyelitis (EAE) model, ITA induces critical metabolic changes by inhibiting MAT and IDH1/2 enzyme activity, thereby suppressing glycolysis and OXPHOS in Th17 and Treg polarized T cells. Adoptive transfer of ITA-treated Th17 polarized T cells resulted in improved EAE [110].

#### Modulating OXPHOS to enhance Treg function

OXPHOS is integral to immune cell function, especially in CD8^+^ T cells and Tregs. Modulating OXPHOS to enhance Treg function could provide a novel strategy to promote immune tolerance and suppress pathogenic T cell responses [78]. OXPHOS strengthens apoptosis resistance and sustains Th17 persistence by preventing the degradation of apoptosis-regulating factors through mitochondrial autophagy [111]. In activated T cells, the enzyme methylene tetrahydrofolate dehydrogenase 2 (MTHFD2) modulates signaling pathways that drive cell proliferation and inflammatory cytokine production [112]. Researches suggest that inhibiting MTHFD2 shifts the metabolic program from glycolysis to OXPHOS, resulting in diminished mTORC1 signaling [113]. Moreover, MTHFD2 inhibition increases Foxp3 expression in Th17 and Tregs under low TGF-β conditions, inducing a metabolic reprogramming of Th17 towards a Treg-like phenotype [83].

In IBD and GvHD, CD226-deficient Tregs modulate the transition from OXPHOS to glycolysis via the AMPK/mTOR/Myc pathway. The use of 10058-F4, a specific inhibitor of MYC-MYC associated factor X (MAX) heterodimerization, effectively restored the impaired function of CD226-deficient Tregs in IBD model [114]. Additionally, proteomic analysis demonstrated that chemical activation of CD4^+^ T cells by the drug-like ClpP agonist NCA029 inhibits OXPHOS and Th17 cell differentiation in vitro, thereby alleviating symptoms of IBD [115]. Somatostatin (SST) ameliorates EAE by reducing mitochondrial respiration and downregulating Th1 and Th17 cell populations through GSK3 activation mediated by SSTR3 [116].

#### FAO regulation for immune tolerance

FAO is integral to amplifying T cell effector functions and preserving membrane integrity. In autoimmune diseases, hyperactive Teffs significantly drive disease progression. Wang et al. targeted FAO regulation by leveraging Zfp335 to directly regulate FHadha, thereby controlling effector Treg differentiation and promoting immune tolerance [117]. G9a influences CD4^+^ T cell lipid biosynthesis by regulating the reduction of H3Kme2 levels [118]. In IBD, G9a inhibition disrupts transcriptional control of lipid synthesis-related genes, leading to elevated intracellular cholesterol levels in T cells, which fosters Treg expansion and mitigates intestinal inflammation [119]. Pdcd1-deficient mice develop colitis, and FAO inhibition effectively restores IL-22 production in Pdcd1-deficient T cells, reducing inflammatory responses [120]. In Crohn’s disease, CD4^+^ T cells upregulate intracellular NF-κB pathway activation, and treatment with etomoxir and ranolazine reverses heir anti-apoptotic and pro-inflammatory phenotypes [121].

Obesity is associated with metabolic dysregulation and persistent inflammation [122]. CD4^+^ T cells in obese mice display elevated FAO activity, marked by upregulated expression of Cpt1a and Goliath, which intensifies activation of the NF-AT pathway. This metabolic shift amplifies T cell glycolysis, contributing to excessive activation and subsequent inflammation [123]. In an obese mouse model, Hao et al. employed the Goliath-specific inhibitor DC-Gonib32 to target the FAO-glycolysis metabolic axis in CD4^+^ T cells, effectively reducing inflammation [124].

### Targeting metabolic reprogramming in cancer immunotherapy

Metabolic reprogramming has garnered significant attention in cancer immunotherapy, as it influences the function of tumor-infiltrating T cells. These cells often undergo metabolic adaptations that impair their ability to mount an effective immune response in the TME [125]. By targeting metabolic pathways, it is possible to enhance T cell function and improve the efficacy of cancer immunotherapies.

#### Enhancing CD8^+^ T cell function

CD8^+^ T cells are critical for the eradication of tumor cells. The transition to aerobic glycolysis is a key hallmark of effector CD8^+^ T cell activation [126]. In CD8^+^ Tm cells, phosphoenolpyruvate carboxykinase (Pck1) is significantly upregulated, facilitating the biosynthesis of G6P [127]. Pck1 serves as a central metabolic regulator, coordinating glycolysis, the TCA cycle, and gluconeogenesis [128]. The Pck1-glycogen-PPP axis forms a critical metabolic network in CD8^+^ Tm cells. In murine tumor models, blocking Pck1-mediated glycogen synthesis through intraperitoneal GPI injection or oral 3-MPA administration accelerates tumor growth [129]. Thus, enhancing Pck1 expression in Tm cells may improve the efficacy of T cell-based immunotherapies.

The ability of CD8^+^ T cell infiltration in solid tumors is correlated with improved patient survival and enhanced response to immunotherapy. Drugs that promote mitochondrial metabolism can effectively increase the intratumoral infiltration of CD8^+^ chimeric antigen receptor T (CAR-T) cells, thereby augmenting therapeutic efficacy [15]. Single-cell RNA sequencing comparing immune cells infiltrating human cancers and syngeneic tumors in female mice revealed that CD8^+^ T cells express prostaglandin E2 receptors EP4 and EP2 upon TCR activation [24]. This expression leads to the downregulation of Il2ra, thereby reducing OXPHOS and glycolysis. EP2 and EP4 antagonists, AS3385282 and ASP7657, were utilized to upregulate OXPHOS and restore CD8^+^ T cell function [24]. Meteorin-like (METRNL) is a cytokine present in the TME, secreted by CD8^+^ T cells during repeated stimulation. Treatment with the PPARδ agonist GW0742, through the targeting of the METRNL-E2F-PPARδ pathway, reduces the maximal respiratory capacity of METRNL, thereby inhibiting the growth of orthotopic gliomas in mice, as well as flank prostate and colorectal cancer [130].

#### Improving immune checkpoint blockade

Immune checkpoint blockade therapies, such as anti-PD-1/PD-L1 antibodies, have revolutionized cancer treatment. However, many patients fail to respond to these therapies, often due to the metabolic exhaustion of tumor-infiltrating T cells [131]. In melanoma brain metastasis, multi-omics analysis displayed significant enrichment of OXPHOS and the TCA cycle [132]. High-precision predictive models were used to demonstrate that CD8^+^ T OXPHOS cells serve as a critical immune population for evaluating the response to immune checkpoint inhibition (ICI) in melanoma patients [133]. In patients with NSCLC, branched-chain amino acid (BCAA) supplementation synergizes with anti-PD-1 therapy to enhance glucose uptake, glycolysis, and OXPHOS through a FoxO1-dependent mechanism, thereby augmenting the effector function of CD8^+^ T cells and promoting antitumor immunity [134].

#### Metabolic reprogramming in adoptive cell therapy

In adoptive cell therapy (ACT), Tmems are integral for their metabolic transition from aerobic glycolysis to FAO [135]. Saibil et al. revealed that activation of PPARα and PPARδ/β pathways in CD8^+^ T cells through the PPAR agonist GW501516 significantly upregulates Cpt1a, the rate-limiting enzyme for FAO [33]. This metabolic shift promotes the sustained viability and functional potency of activated CD8^+^ T cells in ACT models.

CAR-T therapy has revolutionized cancer treatment, while its efficacy against solid tumors remains limited, partly due to the hostile TME and metabolic constraints [136]. Serotonin (5-HT)-mediated serotonylation of glyceraldehyde-3-phosphate dehydrogenase (GAPDH) drives the metabolic shift to glycolysis in CD8^+^ T cells, enhancing anti-tumor immunity. Adoptive transfer of CAR-T cells overexpressing tryptophan hydroxylase 1, which enhances 5-HT production, induces a robust anti-tumor response [137]. Stable overexpression of the GLUT1 in primary human CAR-T cells enhances the secretion of pro-inflammatory cytokines, improves glucose uptake, and promotes T cell expansion and cytolytic capacity [138]. In preclinical models of ALL, RCC, and GBM, this innovative engineering strategy markedly diminished tumor burden [139]. Enasidenib (ENA), a non-competitive inhibitor of the wild-type IDH2 homodimer, reduces oxidative decarboxylation in both the TCA cycle and glycolysis, thereby redirecting glucose metabolism towards PPP. Under nutrient-limited conditions, ENA confers antioxidant protection to CAR-T cells, mitigating their exhaustion and enhancing their metabolic adaptability [140]. Additionally, overexpression of IL-10 enhances OXPHOS in CAR-T cells, improves mitochondrial function, and thereby helps control tumor recurrence [141].

#### Impact of mechanical stress on T cell metabolism

Mechanical forces within the TME, including extracellular matrix (ECM) stiffness and fluid shear stress, significantly influence T cell metabolism and differentiation [142]. ScRNA-seq and TCR sequencing analyses revealed that ECM viscoelastic properties directly modulate T cell phenotypes [143]. T cells cultured in rapidly relaxing matrices exhibit elevated expression of memory markers [144]. By sensing the increased mechanical stiffness of the substrate, Treg induction is enhanced and dependent on OXPHOS. Matrix stiffness in TI-Tregs activates YAP, which enhances mitochondrial OXPHOS by upregulating leucyl-tRNA synthetase 2 (Lars2). Consequently, the combination of a low-leucine diet and YAP inhibitors synergistically induces mitochondrial dysfunction in TI-Tregs, ultimately suppressing tumor growth [145]. In breast cancer, fibrotic TME is characterized by decreased arginine and increased ornithine levels, which impair the function of CD8^+^ T cells [146]. Therapies aimed at reducing fibrotic stiffness to remodel T cell metabolism present novel opportunities for enhancing immunotherapy.

### Combined targeting therapies of T cell metabolism

Recent research has highlighted the limitations of targeting individual metabolic pathways in effectively suppressing pathological immune responses. As a result, combined metabolic targeting strategies are gaining traction as a more effective method to modulate T cell metabolism and control inflammation in autoimmune diseases. Evidence shows that dual inhibition of glycolysis and OXPHOS offers superior regulation of T cell metabolic reprogramming. For example, the concurrent use of 2-DG and the OXPHOS inhibitor metformin has been demonstrated to simultaneously suppress glycolysis and OXPHOS in Th17 cells, leading to a marked reduction in inflammatory responses in autoimmune disease models [108].

Targeting both glycolysis and amino acid metabolism enables precise control over distinct T cell subsets. For instance, inhibiting fatty acid synthesis inhibitors with agents like Etomoxir, combined with glucose metabolism inhibitors such as 2-DG, effectively inhibits Teff proliferation and inflammatory cytokine production, while enhancing Treg differentiation and function [3]. This combined metabolic approach has demonstrated considerable therapeutic potential in autoimmune disease models, including IBD and multiple sclerosis [123]. Additionally, the combination of glutamine metabolism inhibitors (e.g., DON) with cholesterol metabolism inhibitors (e.g., statins) effectively blocks Th17 differentiation and stabilizes Tregs stability, leading to a notable reduction in disease severity in EAE models [92].

## Conclusion and future perspectives

Research on T cell metabolism provides new insights into its role in inflammatory responses and immune regulation. Evidence indicates that distinct T cell subsets rely on different energy and biosynthetic pathways to meet their specific functional requirements. Metabolic reprogramming enables T cells to adapt to diverse inflammatory environments and carry out specialized immune functions, opening avenues for innovative immunotherapeutic approaches. Modulation of T cell metabolism and differentiation allows for precise enhancement or suppression of specific T cell subset activities, offering more targeted immune interventions in the treatment of inflammatory diseases and cancer. This evidence underscores the potential of metabolic pathway regulation for clinical applications.
